# Unified pH Measurements of Ethanol, Methanol, and Acetonitrile, and Their Mixtures with Water

**DOI:** 10.3390/s21113935

**Published:** 2021-06-07

**Authors:** Lisa Deleebeeck, Alan Snedden, Dániel Nagy, Zsófia Szilágyi Nagyné, Matilda Roziková, Martina Vičarová, Agnes Heering, Frank Bastkowski, Ivo Leito, Raquel Quendera, Vítor Cabral, Daniela Stoica

**Affiliations:** 1 DFM A/S, Kogle Allé 5, 2970 Hørsholm, Denmark; asn@dfm.dk; 2 Government Office of the Capital City Budapest (BFKH), Németvölgyi út 37-39, 1124 Budapest, Hungary; nagy.daniel2@bfkh.gov.hu (D.N.); nagyne.szilagyi.zsofia@bfkh.gov.hu (Z.S.N.); 3 Czech Metrology Institute (CMI), Okružní 31, 63800 Brno, Czech Republic; mrozikova@cmi.cz (M.R.); mvicarova@cmi.cz (M.V.); 4 Faculty of Mechanical Engineering, Slovak University of Technology, Namestie Slobody 17, SK-81231 Bratislava 1, Slovakia; 5 Physikalisch-Technische Bundesanstalt (PTB), Bundesallee 100, 38116 Braunschweig, Germany; agnes.heering@ut.ee (A.H.); frank.bastkowski@ptb.de (F.B.); 6 Institute of Chemistry, University of Tartu (UT), Ravila Street 14a, 50411 Tartu, Estonia; ivo.leito@ut.ee; 7 Instituto Português da Qualidade (IPQ), R. António Gião, 2, 2828-513 Caparica, Portugal; rquendera@ipq.pt (R.Q.); vcabral@ipq.pt (V.C.); 8 Laboratoire National de Metrologie et d’Essais (LNE), 1 rue Gaston Boissier, 75015 Paris, France; daniela.stoica@lne.fr

**Keywords:** pH_abs_, ionic liquid salt bridge, commercial glass electrodes, water–alcohol mixture, non-aqueous pH

## Abstract

Measurement of pH in aqueous-organic mixtures with different compositions is of high importance in science and technology, but it is, at the same time, challenging both from a conceptual and practical standpoint. A big part of the difficulty comes from the fundamental incomparability of conventional pH values between solvents (^s^pH, solvent-specific scales). The recent introduction of the unified pH (pH_abs_) concept opens up the possibility of measuring pH, expressed as ${\mathrm{pH}}_{\mathrm{abs}}^{H_{2}O}$, in a way that is comparable between solvent, and, thereby, removing the conceptual problem. However, practical issues remain. This work presents the experience of the authors with measuring ${\mathrm{pH}}_{\mathrm{abs}}^{H_{2}O}$ values in mixtures of methanol, ethanol, and acetonitrile, with water, but without the presence of buffers or other additives. The aim was to assigned ${\mathrm{pH}}_{\mathrm{abs}}^{H_{2}O}$ values to solvent–water mixtures using differential potentiometry and the ‘pH_abs_-ladder’ method. Measurements were made of the potential difference between glass electrodes immersed in different solutions, separated by an ionic liquid salt bridge. Data were acquired for a series of solutions of varying solvent content. This work includes experiences related to: a selection of commercial electrodes, purity of starting material, and comparability between laboratories. Ranges of ${\mathrm{pH}}_{\mathrm{abs}}^{H_{2}O}$ values for selected compositions of solvent–water mixtures are presented.

## 1. Introduction

The familiar pH scale applies to aqueous solutions [1,2] with metrological traceability ensured only for ionic strength, *I*, below 0.1 mol kg^−1^. Measurements of acid-base properties in non-aqueous solvents, and solvent–water mixtures, can be realized using the same definition as aqueous solutions: pH = −log a_H_, where a_H_ is the activity of protons in a given solvent or solvent–water mixture [3,4]. However, in this case, it is critical to point out that each non-aqueous solvent, including different ratios of solvent–water mixtures, has its own, solvent-specific, pH scale, termed ^s^pH, where the s superscript denotes the solvent (or solvent–water mixture). The ^s^pH window is fixed by the autoprotolysis constant of a given solvent.

Over the years, there have been several requests from industry, such as cosmetic, chemical, or printing, and other communities involved in, for example, control of bioethanol, for the development of a reliable metrological infrastructure for non-aqueous media, including appropriate ^s^pH standards. This has turned out to be a daunting task, as each solvent–water mixture composition requires the existence of a reliable set of buffer reference material(s) meeting the specifications previously laid out in reference [3].

Conventional, aqueous pH is measured using a potentiometric method involving an internal reference electrode in ionic contact with a glass electrode (GE) as the pH sensor, and an external reference electrode (RE, usually a Ag/AgCl electrode), where the GE is immersed in the solution under test and the RE is immersed in a reference solution of a known, consistent composition (commonly 3 M KCl (aq)). The reference solution is separated from the solution under test by a porous diaphragm. Routinely, both electrodes are combined into a single probe, known as a combination pH electrode. The pH electrode output is the difference in electric potential between the GE and RE. This measurement is done in two steps. First, the electrode array is calibrated using standard buffers of known pH as to obtain the pH dependence of the potential difference (calibration line) using the Nernst equation. Secondly, pH of the solution under test is measured via conversion of the measured potential difference using the calibration line. During measurement the reference, or filling, solution of a commercial pH electrode comes into contact with the solution under test, creating a liquid junction potential (LJP), also termed the diffusion potential. The error due to the residual liquid junction potential (RLJP), defined as the difference in LJPs between measurements with the standard buffer solutions used for calibration and the solution under test, represents the most severe limitation of using this potentiometric method for pH measurements, especially for measurement of ^s^pH. The advantage of having a RE in a known filling solution is having a consistent reference potential (i.e., anchoring point), rather than only measuring a potential difference without any anchoring point on the potential scale. Additionally, bringing the RE into contact with the solution under test will change the potential in unknown ways, and may result in unfavorable interactions between the electrode and solution. The sign and magnitude of the RLJPs are most often unknown, and depend on multiple factors, including solvent composition (e.g., solvent–water ratio), ionic strength, and temperature. To minimize RLJPs, the pH electrode should ideally be calibrated using solutions of identical solvent composition to the solution under test, so called matrix matching, allowing measurement of pssH (where the subscript s denotes that the electrode has been calibrated in solvent S) [5,6]. 

Largely due to the lack of many ^s^pH buffers, in routine applications commercial pH electrodes are calibrated using dilute aqueous pH buffers, and measurements of pwsH (where the subscript w denotes that the electrode has been calibrated using aqueous buffers) in solvent S are performed. Manufacturers of commercial pH electrodes intended for these applications [2,7,8,9,10] do caution that pwsH measurements are not on the aqueous pH scale. Indeed, metrologically, these types of measurements are not traceable to the definition of ^s^pH nor pH. Such measurements often show poor reproducibility between nominally identical solvents and pH electrodes [11]. 

The pwsH is an operationally defined measurand, i.e., the result is defined by the measurement procedure, including the electrode employed. Since operationally defined methods are very sensitive to variations in the procedure (e.g., fuel ethanol [11]), any changes to the method require a concomitant change in the existing specifications. This is particularly true of bioethanol fuel for which several standards exist in the context of quality assessment [12,13,14,15]. pwsH  measured in bioethanol fuel over a range of compositions (anhydrous and hydrous ethanol) is given, somewhat misleadingly, the name pHe.

To overcome this untenable situation, in 2010 and building on prior work [16,17], Himmel et al. [18] proposed a unified pH scale (pH_abs_) employing a universal reference state, rather than solvent-specific reference states, allowing the placement of all pH_abs_ measurements in diverse solvents, including solvent–water mixtures, on the same scale. Subsequently, the shifted ${\mathrm{pH}}_{\mathrm{abs}}^{H_{2}O}$ scale was proposed [19], permitting the expression of pH_abs_ values on a scale aligned with the well-known aqueous pH scale, i.e., aqueous pH 7 is equivalent to ${\mathrm{pH}}_{\mathrm{abs}}^{H_{2}O}$ 7. Suu et al. [19] demonstrated the practical realization of ${\mathrm{pH}}_{\mathrm{abs}}^{H_{2}O}$ measurements via differential potentiometry. In the most recent version of this method [20] GEs are immersed in two solutions of differing composition separated by an ionic liquid salt bridge (ILSB), triethylamylammonium bis((trifluoromethyl)sulfonyl)imide [N_2225_][NTf_2_]. It has been demonstrated [21,22,23] that this ILSB has several advantages, including: the elimination of the LJP between the two solutions, slower mixing kinetics between IL and organic solvent compared to the more common 3 M KCl (aq) salt bridge, and allowing the assignment of the contribution to uncertainty of the LJP without extra-thermodynamic assumptions. The GE employed were half-cells (vs. combination pH electrodes, which include a RE) and were of a special design, without inner filling solutions. These electrodes consist of a solid-contact between the glass membrane and the internal sensor [19,24], and are referred to as solid-contact glass electrodes (SCGE). The use of half-cells eliminates the possibility of interactions between the inner filling solution and solution under test [20,25]. However, the SCGE used here is not commercially available on a large scale. Similar advantages may be seen in other commercial half-cell electrodes for ${\mathrm{pH}}_{\mathrm{abs}}^{H_{2}O}$ measurements [20]. However, differences between commercial electrodes have been noted when measuring solvent-specific pHe in anhydrous and hydrous bio-ethanol according to ASTM D6423-14 [26,27], as well as buffered methanol–water and ethanol–water solutions [28]. The suitability of various half-cell and combination pH electrodes for ${\mathrm{pH}}_{\mathrm{abs}}^{H_{2}O}$ measurements of mixtures of water with ethanol, methanol, and acetonitrile are investigated in this work.

Ethanol, methanol, acetonitrile, and their mixtures with water, are chosen as examples. Ethanol is selected due to its use as bioethanol in fuel applications. Although methanol and acetonitrile are selected as they are the two solvents used most extensively in liquid chromatography separation [29]. A prior European metrology joint research project (BIOREMA) organized a comparison on a bioethanol reference material, including the assessment of pHe according to methods specified in various standards [30], showed poor comparability of results. Although individual ^s^pH results have been published for methanol and acetonitrile [4,31,32], the evaluation of the reliability of these results over a wider composition range are missing from the literature. Although applications of methanol and acetonitrile to liquid chromatography are combined with buffering agents [29], the use of ethanol in biofuels is un-buffered [33]. Here, measurements with un-buffered solutions, without the addition of other spectator ions, are examined. Particularly, this work sets out to ascertain if commercial pH electrodes (half-cell GE or combination electrodes) can be employed to perform ${\mathrm{pH}}_{\mathrm{abs}}^{H_{2}O}$ measurements by means of differential potentiometry, incorporating an ILSB. This assessment is made by comparison to SCGE measurements. EMPIR project UnipHied (17FUN09) [34] has the goal to develop metrological basis for practical ${\mathrm{pH}}_{\mathrm{abs}}^{H_{2}O}$ measurements. Several national metrology institutes (NMI), participants in the EMPIR project UnipHied, undertook these measurements. Measurement redundancy among NMIs is key to ensuring that the method, and its quality, are completely understood. Participants found determination of ${\mathrm{pH}}_{\mathrm{abs}}^{H_{2}O}$ values in solvent-water mixtures challenging, and demonstrated poor overlap between reported results. This work includes a discussion of these challenges, as a guide for practitioners wishing to implement routine ${\mathrm{pH}}_{\mathrm{abs}}^{H_{2}O}$ measurements. 

## 2. Materials and Methods

All institutes performed differential potentiometric measurements, with glass electrodes and employing an ionic liquid salt bridge (ILSB), based on Cell I:

GE|Solution 1||ILSB||Solution 2|GE,

where || indicates a liquid junction. 

Ionic liquid (C_13_H_26_F_6_N_2_O_4_S_2_, [N_2225_][NTf_2_]) was obtained from Iolitec GmbH (Heilbronn, Germany; courtesy of Dr V. Radtke, University of Freiburg). Solid-contact glass (half-cell) electrodes (SCGE) (Izmeritelnaya Tekhnika EST-0601 [24]) from the same production batch were purchased and distributed to all institutes. SCGEs have previously been proposed as the preferred electrodes for the differential potentiometry measurements enabling calculation of ${\mathrm{pH}}_{\mathrm{abs}}^{H_{2}O}$ values [19,25]. In addition to SCGEs, institutes purchased commercial combination and half-cell GEs, these are summarized in Table 1. All electrodes were stored in the storage solutions provided by the manufactures or in the absence of such storage solutions, in aqueous buffers.

Each institute made measurement on at least two aqueous pH buffers, and a number of organic solvents: methanol, acetonitrile, and ethanol, and their mixtures with water. Potential difference measurements were made between a pair of GEs immersed into two solutions separated by an ILSB, as described in Heering et al. [25]. All measurements were made under thermostating conditions, at 25.0 °C. Compiled potential difference measurements and assigned aqueous pH buffer values (anchor values) were used to calculate ${\mathrm{pH}}_{\mathrm{abs}}^{H_{2}O}$ values using the “pH ladder” method [19,25]. Measurements were done in quiescent solutions. Real time measurements in flow conditions cannot be done with the current measurement method. The exact procedure, including solution preparation, signal treatment, and instrumentation employed at each institute is detailed in the Supplementary Information.

## 3. Results

Tabulated ${\mathrm{pH}}_{\mathrm{abs}}^{H_{2}O}$ data, at 25.0 °C, are presented for the commercial GE and SCGE employed at each institute (see Table 1) in the Supplementary Information. Reference pH values of aqueous pH buffers were employed as anchoring values, their identities are specified for each table. 

**Figure 1:**
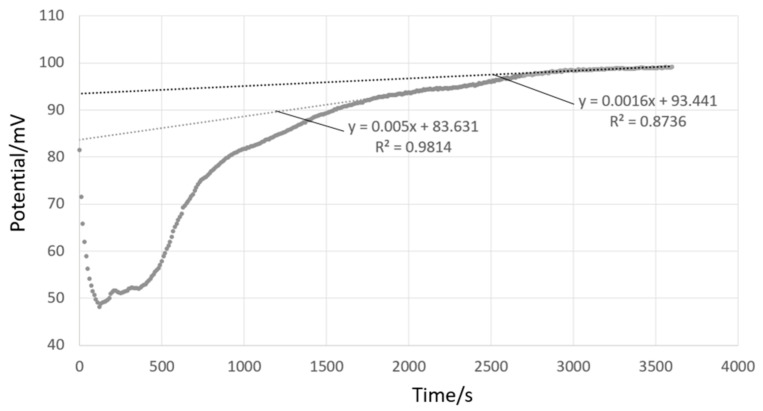
Potential difference measured at DFM with Cell I using 2 SCGE (electrode A) between an ethanol mixture (70 wt% ethanol—30 wt% water) and an acetonitrile mixture (76 wt% acetonitrile—24 wt% water). The final 30 min of data are divided into two 15 min increments, and the extrapolation to t = 0 is shown for each increment.

Transposing the procedure developed for aqueous solutions, the potential difference values (Δ*E*) for each solution pair were evaluated by treating 30 min of data, acquired between t = 1800 s and t = 3600 s. Figure 1 shows an example acquired using two SCGE electrodes (electrode A, Table 1) placed in 70 wt% ethanol—30 wt% water and 76 wt% acetonitrile—24 wt% water, respectively. Solutions were simultaneously added to the two measurement chambers, bringing the solutions into contact with the ILSB. Solutions were previously temperature-equilibrated at the room temperature of the laboratory and were introduced into the thermostated measurements chambers at 25.0 °C. Instead of the recommended conditioning of the electrode in the solvent to be measured [35], the measurement time was increased. As such, the first 30 min of data are expected to include the response of (a) the solutions coming into equilibrium with the ILSB, (b) temperature increasing to 25.0 °C, and (c) stabilization of glass membrane.

In this example, the Δ*E* signal increased towards a more steady drift rate, observed in the final 30 min of data acquisition, corresponding to 180 points taken at 10 s intervals. Δ*E* was evaluated by averaging, referred to as ‘averaging’, over these final 180 points, giving 96.71 mV, with a standard deviation ($\sigma_{\Delta E})$ of 2.07 mV. Linear extrapolation (y =m*t + b) to t = 0 was also employed to evaluate Δ*E*, referred to as ‘extrapolation’, giving: 86.27 mV, the standard deviation of the intercept, $\sigma_{b}$, being 0.20 mV. However, this standard deviation does not reflect the change seen between t = 1800 s and t = 3600 s. For more representativeness, the final 30 min of data were divided into two 15 min increments, and the respective intercepts were determined as: 83.63 mV and 93.44 mV, as illustrated in Figure 1. The uncertainty of Δ*E* evaluated by extrapolation, $u_{\Delta E}$, equals 5.66 mV, considering the variability between the two extrapolated values and assuming a rectangular distribution.

However, for all organic solvents, and their mixtures with water, it was observed that, in the absence of buffering agent, the system illustrated by Cell I includes multiple interactions, including between the GE and solutions under test, as well as between solutions under test and the ILSB. This system does not reach equilibrium, which would be indicated by a steady Δ*E* with time, within the time of measurement. The measurement was limited in time due to the gradually increasing miscibility of the ILSB in the solvent-water mixtures. The ‘averaging’ Δ*E* evaluation method allows a more consistent snapshot of the $\Delta {\mathrm{pH}}_{\mathrm{abs}}$ following temperature equilibration at 25.0 °C. Therefore, the averaging Δ*E* evaluation method was selected for presentation of ${\mathrm{pH}}_{\mathrm{abs}}^{H_{2}O}$ data.

Using electrode A (SCGE), ${\mathrm{pH}}_{\mathrm{abs}}^{H_{2}O}$ values were measured using nominally identical electrodes (same manufacturing batch) and equivalent setups (Cell I) at participating institutes. As these measurements are made using largely similar electrodes, setups, and solution compositions, the ${\mathrm{pH}}_{\mathrm{abs}}^{H_{2}O}$ values, which were calculated using the ‘averaging’ data evaluation method, have been combined. The equivalence of ${\mathrm{pH}}_{\mathrm{abs}}^{H_{2}O}$ measurements performed at participating institutes has previously been demonstrated in aqueous solutions [25,36] and buffered ethanol-water mixtures [36]. In buffered water–ethanol mixtures, results obtained at PTB and DFM for equimolal phosphate buffered 50–50 wt% ethanol–water, as well as UT and DFM for ammonium formate buffered anhydrous ethanol, showed a high degree of consistency [36]. This suggested that the method of ${\;\mathrm{pH}}_{\mathrm{abs}}^{H_{2}O}$ evaluation by differential potentiometry was robust enough to be set up and used for even more challenging media, such as unbuffered solvent–water mixtures.

**Figure 2:**
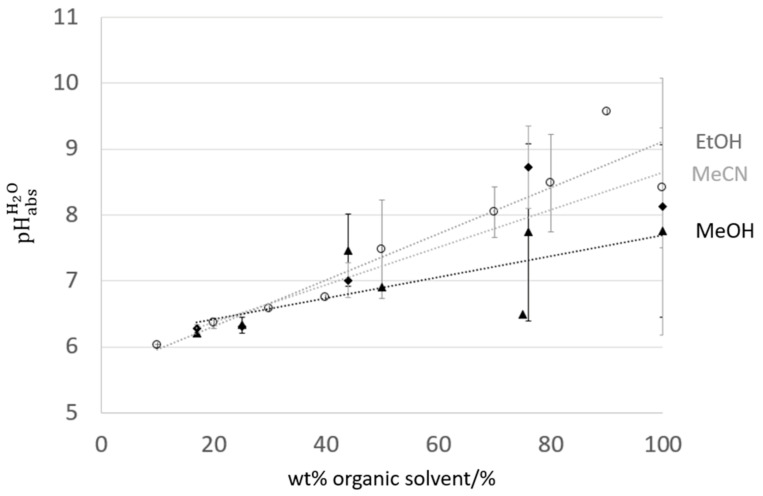
${\mathrm{pH}}_{\mathrm{abs}}^{H_{2}O}$ values for mixtures of ethanol (EtOH, ○), acetonitrile (MeCN, ◆), and methanol (MeOH, ▲) with water, measured at institutes using SCGEs. Error bars are calculated using Equation (1), and represent the distribution of values measured at different institutes.

In Figure 2, ${\mathrm{pH}}_{\mathrm{abs}}^{H_{2}O}$ values measured using SCGE (electrode A) and calculated using the averaging potential difference method are presented for mixtures of water and methanol, ethanol, and acetonitrile. Uncertainties were assigned for each solvent–water ratio measured at n ≥ 2 institutes, using the equation below, assuming a rectangular uncertainty distribution.

(1) $$u=\frac{E_{max}-E_{min}}{\sqrt{3}},$$

where *E_max_* is the largest reported, and *E_min_* is the smallest reported ${\mathrm{pH}}_{\mathrm{abs}}^{H_{2}O}$ value for a given solvent–water mixture.

The ${\mathrm{pH}}_{\mathrm{abs}}^{H_{2}O}$ values largely converge at lower solvent content, extrapolated value at ‘pure’ water (0 wt% solvent) level provided ${\mathrm{pH}}_{\mathrm{abs}}^{H_{2}O}$ values between 5.6 to 6.1 (5.85 ± 0.26).

### 3.1. ^s^pH Values of Water–Solvent Mixtures 

For each series of solvent–water mixtures, literature ^s^pH values are presented between pure water and pure solvent. Many of these values are calculated using the formulas presented in [37]. This simplified formula, for determination of autoprotolysis constants (p*K*_ap_) in solvent–water mixtures, allows an estimation of neutral ^s^pH (solvent-specific scales) for different ratios (psH=½pKap). In pure solvent (HS), the autoprotolysis reaction is given by:

$$\mathrm{HS}\;=H^{+}+S^{-}$$

For a mixture between water (H_2_O) and an organic solvent (HS), p*K*_ap_ is determined as a function of the mole fraction of solvent ($x_{\mathrm{HS}}$) and water ($x_{H_{2}O}$ = 1 − $x_{\mathrm{HS}}$):

(2) $$\begin{array}{cc} pK_{\mathrm{ap}}=x_{H_{2}O} & \times p\left(K_{a,H_{2}O\left(H_{2}O\right)}+\frac{K_{H_{2O}}\times K_{\mathrm{HS}}}{K_{a,H_{2}O\left(H_{2}O\right)}}\right)\\ & +x_{\mathrm{HS}}\times p\left(K_{a,{\;H}_{2}O\left(\mathrm{HS}\right)}+\frac{K_{H_{2O}}\times K_{\mathrm{HS}}}{K_{a,H_{2}O\left(\mathrm{HS}\right)}}\right)-log\;x_{H_{2}O}-log\;x_{\mathrm{HS}} \end{array}$$

where $K_{H_{2}O}$ and $K_{\mathrm{HS}}$ are the autoprotolysis constant of pure water and solvent, respectively, and $K_{a,H_{2}O\left(H_{2}O\right)}$ and $K_{a,H_{2}O\left(HS\right)}$ are parameters tabulated for selected solvents in Table III of [37]. 

The mole fraction of solvent ($x_{\mathrm{HS}}$) is converted to weight percentage (wt%):

(3) $$wt\%=100\left(\frac{x_{\mathrm{HS}}\times M_{\mathrm{HS}}}{x_{H_{2}O}\times M_{H_{2}O}+x_{\mathrm{HS}}\times M_{\mathrm{HS}}}\right)$$

where $M_{H_{2}O}$ and $M_{\mathrm{HS}}$ are the molecular weights of water and solvent, respectively.

**Figure 3:**
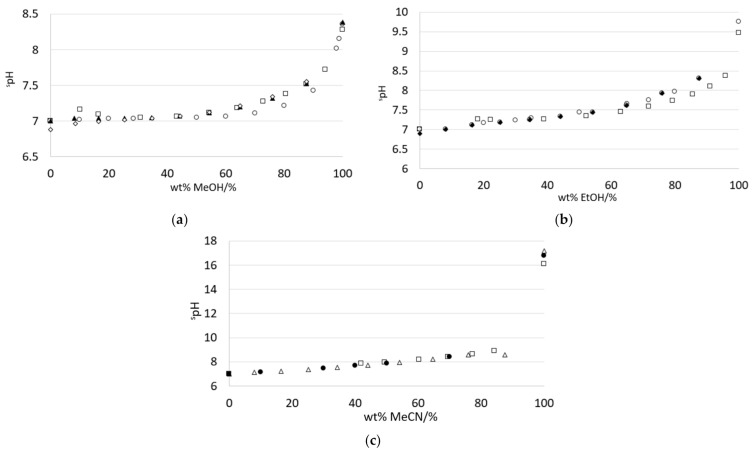
Theoretical ^s^pH values for three water–solvent mixture systems: (**a**) water–methanol, (**b**) water–ethanol, and (**c**) water–acetonitrile. (**a**) water–methanol mixtures: (○) [38], (◊) Table 1 in [39], (▲) Table 1 in [40], and (□) calculated according to [37]. (**b**) water–ethanol mixtures: (○) various literature sources collected in [41], (◆) Table 1 in [42], and (□) calculated according to [37]. (**c**) water–acetonitrile mixtures: (Δ) Table 1 in [43], (●) Table 6 in [44], and calculated according to [37].

Figure 3a–c shows the theoretical ^s^pH values for the three analyzed water–solvent mixtures. Theoretical ^s^pH variation as a function of the solvent composition shows the same profile for various literature sources. However, starting at ~50 wt% methanol for water–methanol, ~65 wt% ethanol for water–ethanol, and ~90 wt% acetonitrile for water–acetonitrile, different literature sources assigned slightly different ^s^pH values.

### 3.2. $pH_{abs}^{H_{2}O}$ Values of Water–Organic Solvent Mixtures

**Figure 4:**
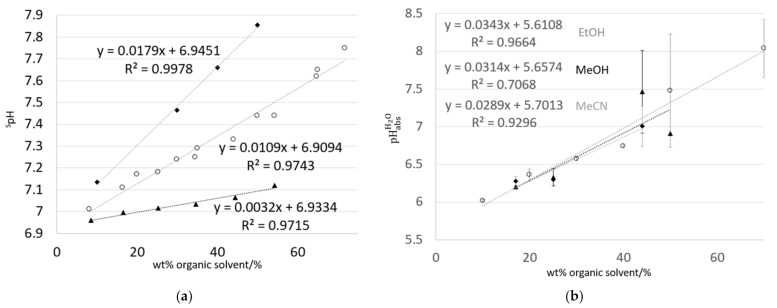
Between >0 wt% and ≤70 wt% organic solvent: (**a**) Literature ^s^pH values for mixtures of water with ethanol (○) [41], methanol (▲) [39], and acetonitrile (◆) [44], and (**b**) ${\mathrm{pH}}_{\mathrm{abs}}^{H_{2}O}$ values, measured at institutes using SCGEs. Wt% solvent is chosen based on data available from the water–organic mixtures studied here: 10 wt%–70 wt% ethanol, 17 wt%–50 wt% methanol, and 17 wt%–44 wt% acetonitrile. Error bars are calculated using Equation (1), and represent the distribution of values measured at different institutes.

There is a change in the regime associated with standard chemical potentials of single ions, including H^+^, reported at >70 wt% ethanol in ethanol–water mixtures [45]. This cutoff (i.e., ≤70 wt% solvent) is extended to both methanol and acetonitrile. Data are presented in the composition range > 0 wt% and ≤70 wt% solvent in Figure 4a. Literature values of ^s^pH are chosen from sources as independent as possible, i.e., not all derived from equations proposed by [37] (see discussion above). For the selected composition range (>0 wt% and ≤70 wt% organic solvent), ^s^pH values increase linearly with solvent content. ${\mathrm{pH}}_{\mathrm{abs}}^{H_{2}O}$ values, measured using SCGE and with potential differences calculated by averaging over 30 min of data, also show a linear increase. Extrapolation to ‘pure’ water (0 wt% organic solvent) give a ${\mathrm{pH}}_{\mathrm{abs}}^{H_{2}O}$ of 5.61 to 5.70 (5.66 ± 0.05). This is consistent with the pH and variability reported for air-equilibrated water [46]. ${\mathrm{pH}}_{\mathrm{abs}}^{H_{2}O}$ values for the same mixtures are shown in Figure 4b.

**Figure 5:**
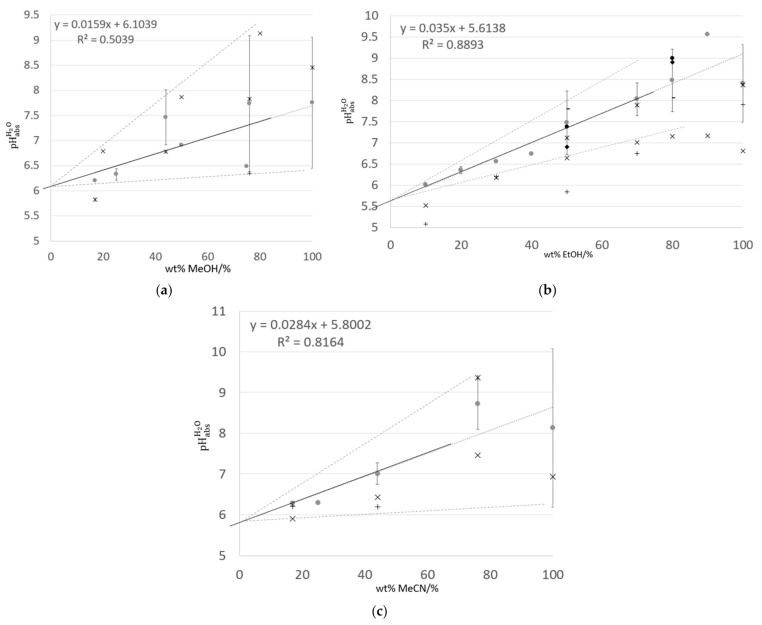
${\mathrm{pH}}_{\mathrm{abs}}^{H_{2}O}$ values measured in different water—organic mixtures: (**a**) water–methanol, (**b**) water–ethanol and (**c**) water–acetonitrile. For the SCGE (●), values are averaged between institutes. Error bars are calculated using Equation (1), and represent the distribution of values measured at different institutes. Solid line is the linear trend line for averaged ${\mathrm{pH}}_{\mathrm{abs}}^{H_{2}O}$ values measured using SCGE as a function of wt% organic content. Dotted lines are included as a guide to the expected variability in ${\mathrm{pH}}_{\mathrm{abs}}^{H_{2}O}$ values, which may be measured using SCGE for different water–organic ratios. (**a**) water–methanol mixtures: potential difference measured using combination (B, × and F, *), half-cell (G, +), and SCGE (A, ●). (**b**) water–ethanol mixtures: potential difference measured using combination (F, ×), half-cell (G, *; H, + and −; I, ◦; J, ◊), and SCGE (A, ●). Values for electrodes H, I, and J are taken from [20]. (**c**) water–acetonitrile mixtures: potential difference measured using combination (F, ×), half-cell (G, * and H, +), and SCGE (A, ●).

Figure 5a–c shows the ${\mathrm{pH}}_{\mathrm{abs}}^{H_{2}O}$ values measured for the three water–solvent mixture systems analyzed using SCGE (electrode A) and various commercial electrodes at a number of institutes.

The relationship between ^s^pH and ${\mathrm{pH}}_{\mathrm{abs}}^{H_{2}O}$ is established through:

(4) pHabsH2O=−ΔtrG⦵H+, H2O→SRTln10+psH

As expected, for water-solvent mixtures, ${\mathrm{pH}}_{\mathrm{abs}}^{H_{2}O}$ values shown in Figure 5 are numerically dissimilar to the theoretical ^s^pH values given in Figure 3. The difference between the two concepts is fixed by the medium effect on the hydrogen ion through its transfer activity coefficient from water to a solvent, S, log γ_tr_$\left(H^{+},{\;H}_{2}O\to S\right)$, where

(5) $$\log\gamma_{\mathrm{tr}}\left(H^{+},{\;H}_{2}O\to S\right)=\frac{\Delta_{\mathrm{tr}}G^{⦵}\left(H^{+},{\;H}_{2}O\to S\right)}{RT\ln10}$$

where S symbolizes a non-aqueous solvent, including different ratios of water–solvent mixtures. log γ_tr_$\left(H^{+},{\;H}_{2}O\to S\right)$ can be seen as an inter-solvent link and is considered a key element in the creation of the unified pH concept [47]. 

Generally, the spread of values measured using identical electrodes (electrode A), seen as error bars in Figure 5, increases with solvent content. For water–methanol solutions (Figure 5a), ${\mathrm{pH}}_{\mathrm{abs}}^{H_{2}O}$ values measured using commercial electrodes show reasonable agreement within the spread reported for electrode A measurements, with the exceptions of 17 wt% methanol using electrode F, and 20 wt% methanol using electrode B. For water–ethanol solutions (Figure 5b), the spread of values measured using identical electrodes (electrode A), is generally larger at ≥50 wt% ethanol. Additionally, ${\mathrm{pH}}_{\mathrm{abs}}^{H_{2}O}$ values do not show the expected increase in value at >70 wt% ethanol. In contrast, the ${\mathrm{pH}}_{\mathrm{abs}}^{H_{2}O}$ values decrease between 90 wt% and 100 wt% ethanol. The spread in ${\mathrm{pH}}_{\mathrm{abs}}^{H_{2}O}$ values reported using SCGE (electrode A) for pure ethanol (100 wt%) are within ±0.8, which represents a higher level of agreement relative to the reproducibility standard deviation of 1.9 reported for pHe in hydrous bioethanol within the inter-laboratory comparison organized within the BIOREMA project [30]. Generally, ${\mathrm{pH}}_{\mathrm{abs}}^{H_{2}O}$ values measured using commercial electrodes are lower than those measured using SCGE (electrode A). For water–acetonitrile solutions (Figure 5c), the ${\mathrm{pH}}_{\mathrm{abs}}^{H_{2}O}$ values measured using commercial electrodes are in poor agreement with those measured using SCGE (electrode A). Commercial electrodes gave systematically lower ${\mathrm{pH}}_{\mathrm{abs}}^{H_{2}O}$ values, with the exception of 76 wt% acetonitrile using Electrode G.

## 4. Discussion

Inter-laboratory comparability of SCGE measurements in un-buffered non-aqueous media (vs. buffered aqueous media [25]) is relatively poor. The poor overlap seen in water–(methanol, ethanol, acetonitrile) mixtures suggests the need for careful solution preparation, storage, and use protocols in order to obtain reference values. A small, but noteworthy, consideration remains the details of the differential potentiometry method, including the need to fill both measurement chambers with identical masses of sample (rather than volumes), and timing the immersion of the pair of electrodes, such that equal forces are experienced on the ILSB by both chambers.

It is likely that the presented spread between institutes includes several influences: the inherent instability of the reading, as shown in Figure 1, which is different on between replicate measurements, which leads to scatter of results both within and between labs. Further influences include: initial purity (including water content) of organic solvent (including bottle to bottle differences), storage conditions and duration, and initial pH of water used in solvent-water mixtures. Further influences may include: differences in electrode construction, including glass composition [26,48] and those associated with interaction between the inner filling solution and the solution under test for combination pH electrodes (electrodes B through F) [46]. 

### 4.1. Solvent

The exact compositions of prepared solvent–water mixtures were not verified, relying on the stated solvent purity of the manufacturers and the masses of solvent and water used in preparation. As such, at the time of data acquisition, the actual composition of each solvent–water mixtures may vary from the stated nominal composition. Methanol, ethanol, and acetonitrile are volatile solvents, which will evaporate at room temperature. From the time that ‘pure’ solvent bottles are opened, their composition will change due to the absorption of water from the air. PTB investigated the influence of initial ethanol purity (as stated by the manufacturer), measuring ${\mathrm{pH}}_{\mathrm{abs}}^{H_{2}O}$ on the same system (Cell I) for mixtures containing 50 wt% and 80 wt% ethanol (Table S9). A 0.4 % change in purity (99.9% and 99.5%) of ethanol translated into changes of ${\mathrm{pH}}_{\mathrm{abs}}^{H_{2}O}$ values that depend on the amount of organic solvent in the mixtures, i.e., the higher the solvent content, the higher the solution sensitivity. Indeed, a change in ${\mathrm{pH}}_{\mathrm{abs}}^{H_{2}O}$ of ~1.3 was calculated for the solutions containing 80 wt% ethanol, in contrast to 0.6 for solutions containing 50 wt% ethanol. These changes may be due to acetic acid impurity in ethanol. Additionally, differences in ${\mathrm{pH}}_{\mathrm{abs}}^{H_{2}O}$ were observed between two different bottles of nominal identical solution (same manufacturer) of 100 % methanol (Table S10).

However, the uncertainty assigned to ${\mathrm{pH}}_{\mathrm{abs}}^{H_{2}O}$ values obtained with SCGE (electrode A)—shown as error bars in Figure 5—is expected to encompass variation in purity of solvent, and initial water pH employed. Additionally, as un-buffered solutions are not expected to show stable ${\mathrm{pH}}_{\mathrm{abs}}^{H_{2}O}$ with (storage) time, the assigned uncertainty takes into consideration the variable conditions and time of storage (between solution manufacture and measurement). Stability issues with storage over several days may arise from continued interaction between the water component of the mixture and atmospheric CO_2_ [46], evaporation of the more volatile component (e.g., alcohol), interaction with the storage vessel material, etc.

### 4.2. Interaction with ILSB

**Figure 6:**
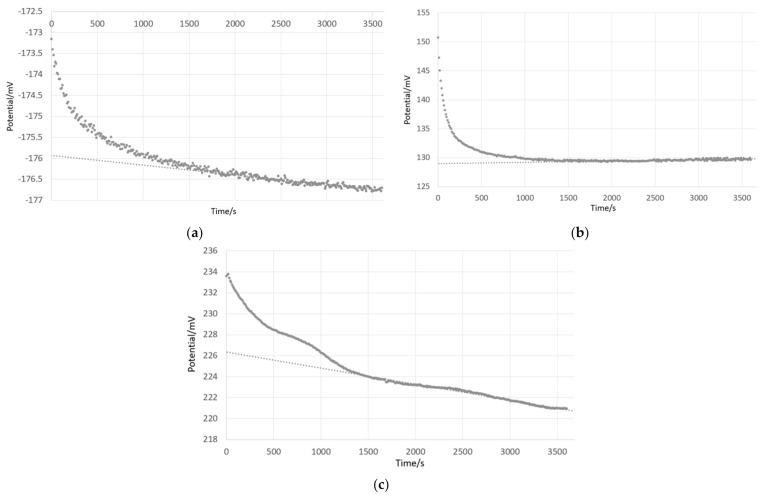
Potential difference measurements using two SCGE (DFM) for (**a**) pH 4.005 and pH 7.000 aqueous buffers, (**b**) pH 4.005 and 76 wt% methanol—24 wt% water, and (**c**) pH 4.005 and 70 wt% ethanol—30 wt% water.

The ILSB is miscible with certain organic solvents, especially at high solvent contents. The gradually increasing miscibility of the ILSB with the solution(s) under test may lead to an unstable junction, which results in an unstable Δ*E* signal over longer time scales, i.e., several hours. Although a stable drift, seen with aqueous buffers as well, potential slope can be observed within 1 h of beginning of measurement. The observed drift is dependent on the two solutions that fill the differential cell. Water–methanol solutions showed low drift (e.g., Figure 6b, 0.8 mV h^−1^), similar to that observed for aqueous buffers (e.g., Figure 6a, −0.8 mV h^−1^), compared with the behavior of water–ethanol mixtures, shown in Figure 6c, for which a slope of −5.5 mV h^−1^ was recorded in the final 30 min of data. This suggests that the ILSB is more stable when in contact with aqueous and water–methanol mixtures. When high organic content solvent-water mixtures are present on both sides of the ILSB, the drift become more pronounced, as shown in Figure 1 for 70 wt% ethanol—30 wt% water measured against 76 wt% acetonitrile—24 wt% water (slope = 13.9 mV h^−1^).

### 4.3. Data Analysis Methodology

As described in the Results section, the data extraction method does make a difference in ${\mathrm{pH}}_{\mathrm{abs}}^{H_{2}O}$ value assigned to each solvent-water mixture. Data evaluated using the extrapolation method generally results in lower ${\mathrm{pH}}_{\mathrm{abs}}^{H_{2}O}$ values. However, the trends presented in Figure 5a–c remain identical. The Δ*E* data used to build the pH_abs_ ladder evaluation, including the number of data input relative to ${\mathrm{pH}}_{\mathrm{abs}}^{H_{2}O}$ values to be output influence the resultant ${\mathrm{pH}}_{\mathrm{abs}}^{H_{2}O}$ values of all solutions included in the ladder. This can be seen for data acquired at DFM, where two ${\mathrm{pH}}_{\mathrm{abs}}^{H_{2}O}$ ladders were constructed (Tables S2 and S3), in addition to the anchoring aqueous buffers, one including data from only ethanol–water solutions, and the second additionally containing data from acetonitrile and methanol solutions. Solutions included in both tables are 50 wt% and 70 wt% ethanol, which show different ${\mathrm{pH}}_{\mathrm{abs}}^{H_{2}O}$ values depending on data used in each ${\mathrm{pH}}_{\mathrm{abs}}^{H_{2}O}$ ladder.

Moreover, replicate measurements performed at individual institutes (identical solutions, electrodes, and setup) showed poor repeatability. Hence, a source of variability between $\;{\mathrm{pH}}_{\mathrm{abs}}^{H_{2}O}$ values, the repeatability of Δ*E* measurements should be included in the overall uncertainty budget for the ${\mathrm{pH}}_{\mathrm{abs}}^{H_{2}O}$ values assigned using the ladder methodology.

### 4.4. Variability between GE

SCGE (electrode A) differential potentiometry have previously been shown to allow the determination of ${\mathrm{pH}}_{\mathrm{abs}}^{H_{2}O}$ values for buffered aqueous solutions described in the [25] and solvent–water [19,20,49,50] mixtures, as well as p*K*_a_ values in acetonitrile [51]. The use of these electrodes in Cell I has been agreed to be the ‘reference’ method for ${\mathrm{pH}}_{\mathrm{abs}}^{H_{2}O}$ value determination [36]. Of more importance in the present work is that all institutes had a pair of electrodes A from the same manufacturing batch, with characteristics as similar as possible, allowing combination of these results between institutes.

Measurements made using the system described by Cell I are quiescent (not stirring or flowing), on low buffer capacity solutions. Variability between a number of commercial GE has been reported in quiescent, low buffering capacity aqueous solutions by Midgley and Torrance [48]. These authors reported bias of up to 0.3 pH-units in purely aqueous systems. Isolating for variability arising solely from the GE component (vs. RE component) of pH electrodes is not straightforward. The liquid junction between the RE, its filling solution, and the solution under test was identified as being the main contributor to inter-electrode variability. This conclusion has been drawn for both low ionic strength (poor buffering capacity) aqueous solutions [46] and buffered alcohol–water mixtures [29]. The signal from the RE component of the combination pH electrodes employed here (electrodes B through F) is not used in the current investigation. However, the filling solution from the RE compartment is designed to leak into the solution under test, changing its composition with time. Any influence of this gradual composition change will be seen much more prominently in poorly buffered solution, than in typical aqueous pH buffers [46]. Consequently, given (i) the difficulty in isolating the signal of the GE component (vs. RE component), and (ii) the effects of the composition of the solution under test, the use of combination glass electrodes for ${\mathrm{pH}}_{\mathrm{abs}}^{H_{2}O}$ measurements of un-buffered water–solvent mixtures is not recommended.

Commercial GEs gave overall lower ${\mathrm{pH}}_{\mathrm{abs}}^{H_{2}O}$ values than measured using electrode A for most compositions of acetonitrile and ethanol based mixtures (Figure 5b,c). This trend was not observed for methanol-water solutions (Figure 5a). Midgley and Torrance [48] proposed that quiescent, low buffering capacity solutions may display lower pH values due to hydroxide ions in solutions, in proximity to the GE membrane, attacking the silicate glass. As this process consumes hydroxide ions, the indicated pH value decreases. This effect requires OH^−^ ions to be present in solution, which may not be the case in pure solvent. Further, this effect would require there to be a significant glass compositional difference between the majority of commercial GEs and the SCGE (electrode A), such that this effect is observed for commercial GE and not for SCGE.

Given the small variation expected in commercial GE glass composition [48], there is no clear explanation as to why different GE half-cells should give different ${\mathrm{pH}}_{\mathrm{abs}}^{H_{2}O}$ values for identical solvent-water mixtures. In this context, it is reasonable to believe that creating and maintaining (with time) identical mixtures is likely the limiting factor in acquiring overlapping ${\mathrm{pH}}_{\mathrm{abs}}^{H_{2}O}$ values for a given nominal solvent-water mixture composition. There is no reason, a priori, that commercial GE half-cell (vs. combination electrodes) cannot be used to perform differential potentiometry measurements for determination of ${\mathrm{pH}}_{\mathrm{abs}}^{H_{2}O}$ values.

### 4.5. Metrological Comparability of $pH_{abs}^{H_{2}O}$ Values

Theoretical evaluation of solvent mixtures up to ~70% solvent composition show linear progression for ^s^pH (assuming ^s^pH = ½ p*K*_ap_) values of mixtures of water with methanol, ethanol, and acetonitrile (Figure 4a). For this range, linear trends are also seen for ${\mathrm{pH}}_{\mathrm{abs}}^{H_{2}O}$. values as a function of increasing solvent content. Graphs of solvent-specific ^s^pH values, primarily derived by theoretical means, assume the pH of ‘pure water’, ^w^pH = 7 (at 25.0 °C). However, pH of water is generally not 7, but lower due to dissolved CO_2_ [46,52,53]. Comparing the trends in ^s^pH and ${\mathrm{pH}}_{\mathrm{abs}}^{H_{2}O}$ with wt% solvent (Figure 4a,b) are then distorted as ^s^pH does not drop much below 7, unlike seen for ${\mathrm{pH}}_{\mathrm{abs}}^{H_{2}O}\;$ for < 40–60 wt% organic solvent. It is worth re-emphasizing that ${\mathrm{pH}}_{\mathrm{abs}}^{H_{2}O}$ values are on the same scale, while each ^s^pH value for a given solvent–water mixture is its own scale, i.e., the ^s^pH scale of 40 wt% ethanol is not the same scale as for 50 wt% ethanol.

### 4.6. $pH_{abs}^{H_{2}O}$ Ranges for Unbuffered Solvent–Water Mixtures

The ${\mathrm{pH}}_{\mathrm{abs}}^{H_{2}O}$ of a given pure solvent, or solvent-water mixture, is likely to exhibit a value range, rather than a singular value, which may be expected for buffered solutions. This is well known for ‘pure’ water (i.e., 0 % solvent content) [52], where the pH depends strongly on the dissolved and dissociated CO_2_ content (acidification of water) [54]. The pH of the water component will change with time due to interaction with atmospheric CO_2_ [46]. Further, measuring the pH of ‘pure’ water (e.g., UPW or DI water) with GE, and various RE, can be challenging [46]. It is commonly recommended to increase the spectator ion concentration to raise the conductivity above 50 µS cm^−1^ [55], in order to make a reliable measurement.

In the case of ethanol–water mixtures, anhydrous and hydrous bioethanol fuel is required to have a solvent-specific pH (pHe) between 6.5 and 9.5 at 25.0 °C [17,33]. This range exists to encompass variations arising from the bioethanol production process, water content (hydrous bioethanol), and presence of additives [33]. Further, a report of ethanol–water mixtures used in the preservation of museum collections has shown a wide dispersion in the pwsH of solutions of identical measured ethanol content [41].

Literature ^s^pH values of methanol–water (Figure 3a) and acetonitrile–water (Figure 3c) mixtures do show some disagreement between values at given wt% solvent, and the ‘pure’ solvent. In light of variability seen in various ^s^pH scales of solvent–water mixtures, it seems unlikely that solvent–water mixtures without additives can be assigned singular reference ${\mathrm{pH}}_{\mathrm{abs}}^{H_{2}O}$ values, although ranges can be assigned.

## 5. Conclusions

The data presented in the paper provide means of understanding the sensitivities and challenges of the ${\mathrm{pH}}_{\mathrm{abs}}^{H_{2}O}$ values for three un-buffered non-aqueous systems, based on the differential potentiometry method between two glass electrodes. The influencing factors can be separated into those relating to the sample itself (preparation including the wt% solvent content, quality of water and pure solvent, stability in time, evaporation, etc.), properties of liquid junction (miscibility and stability of the junction), and materials used (type of electrode, presence of RE and filling solution).

${\mathrm{pH}}_{\mathrm{abs}}^{H_{2}O}$ value ranges are reported for a wide range of compositions of unbuffered mixtures of ethanol, methanol, and acetonitrile with water, at 25.0 °C. Measurements have been carried out by several NMIs. Results obtained using the ‘reference’ method, i.e., using solid-contact glass electrodes (SCGE) have been compared with those obtained using commercial half-cell and combination glass electrodes. Limited systematic difference is observed between commercial half-cell GE and SCGE. Although combination pH electrodes are not recommended for these types of measurements. There is no clear evidence that commercial GE half-cells cannot be used for measurements of ${\mathrm{pH}}_{\mathrm{abs}}^{H_{2}O}$ of solvent–water mixtures by differential potentiometry, with pairs of solutions separated by an ionic liquid salt bridge (ILSB). The presented setup (Cell I) may be taken into use in routine laboratory pH measurements, allowing the placement of ‘pH’ values of solvent–water mixtures onto the same scale. This is in contrast to the current practice of calibrating pH electrodes with aqueous pH buffers, and making measurements in solvent mixtures (pwsH), which only provide indicative values, which lack metrological traceability.

The next step is to do an in-depth uncertainty analysis of the ${\mathrm{pH}}_{\mathrm{abs}}^{H_{2}O}$. Currently there are no standards for ${\mathrm{pH}}_{\mathrm{abs}}^{H_{2}O}$ measurement. One aim of the EURMET project UnipHied is to disseminate the findings of the project to the European measurement infrastructure and relevant standards development organizations to initiate the standardization.

## Figures and Tables

**Table 1:** Glass electrodes employed at each institute.

	Electrode Type	Electrode	Institute
A	SCGE	EST-0601, Izmeritelnaya Tekhnika	CMI; DFM; IPQ; PTB; UT
B	Combination	6.0229.100, Metrohm	BFKH
C		Ross Orion SureFlow, Thermo Fischer Scientific	CMI
D		P11/KJ/LICL, Sentek	CMI
E		Polylyte Plus, Hamilton	DFM
F		Metrohm 6.0269.100	PTB
G	Half-cell	E11M001, Radiometer	DFM
H		6.0150.100, Metrohm	IPQ; PTB ^1^
I		DG300-SC, Mettler-Toledo	PTB ^1^
J		Model 1076-10C, Horiba Scientific/Laqua	PTB ^1^

^1^ Data original presented in [20].

## Supplementary Materials

The following are available online at https://www.mdpi.com/article/10.3390/s21113935/s1, Table S1: BFKH (Electrode B) calculated ${\mathrm{pH}}_{\mathrm{abs}}^{H_{2}O}$ values. Table S2: DFM (Electrode G) calculated ${\mathrm{pH}}_{\mathrm{abs}}^{H_{2}O}$ values—ethanol solutions. Table S3: DFM (Electrode G) calculated ${\mathrm{pH}}_{\mathrm{abs}}^{H_{2}O}$ values. Table S4: IPQ (Electrode H) calculated ${\mathrm{pH}}_{\mathrm{abs}}^{H_{2}O}$ values. Table S5: PTB (Electrode F) calculated ${\mathrm{pH}}_{\mathrm{abs}}^{H_{2}O}$ values. Table S6: CMI (Electrode A) calculated ${\mathrm{pH}}_{\mathrm{abs}}^{H_{2}O}$ values. Table S7: DFM (Electrode A) calculated ${\mathrm{pH}}_{\mathrm{abs}}^{H_{2}O}$ values. Table S8: IPQ (Electrode A) calculated ${\mathrm{pH}}_{\mathrm{abs}}^{H_{2}O}$ values. Table S9: PTB (Electrode A) calculated ${\mathrm{pH}}_{\mathrm{abs}}^{H_{2}O}$ values. Table S10: UT (Electrode A) calculated ${\mathrm{pH}}_{\mathrm{abs}}^{H_{2}O}$ values.
