# Supplementary material for: Unified pH Measurements of Ethanol, Methanol, and Acetonitrile, and Their Mixtures with Water
Source: Sensors (Basel). 2021 Jun 7;21(11):3935. doi: 10.3390/s21113935 (PMC8201227; doi:10.3390/s21113935)
Supplement: Supplementary file 1 [file sensors-21-03935-s001.zip › sensors-1205640-supplementary.pdf]

Supplementary Information

Experimental methods

BFKH

Measures were performed in a thermostated cell (25.0 °C), using two commercial GE (combination electrodes) (Metrohm 6.0229.100) with 2 M LiCl in ethanol filling solution. These were calibrated using aqueous pH buffers (4.008, 6.868, 7.413, and 9.180 at 25 °C, ± 0.01–0.02, BFKH CRMs) versus their respective internal RE (Ag/AgCl). The signal of the GE portion of the combination electrodes were isolated using a BNC to banana plug converter. Potential difference was measured using a Keithley 6430 multimeter. Data was acquired for 2.5 h to 3 h at 3 s intervals. Solutions used in constructing the $pH_{abs}^{H_2O}$ ladder included: three aqueous pH buffers (4.008, 6.868, and 9.18), and 3 water-ethanol solutions (20%, 50%, and 80% ethanol). Water-ethanol solutions were prepared gravimetrically (wt% basis) using anhydrous ethanol (Sigma-Aldrich) and UPW.

Data was analyzed by averaging over several hours of data (averaging), or by extrapolating this data to time zero (extrapolated). $pH_{abs}^{H_2O}$ ladders were constructed using both data analysis methods, using aqueous pH buffers 4.008 and 6.865 as anchoring buffers.

CMI

Measures were performed in a thermostated cell (25.0 °C), using two pairs of commercial GE (combination electrodes): Ross Orion SureFlow, Thermofisher (used in ASTM D6423-14 methodology for measurement in anhydrous ethanol) and P11/KJ/LICL pH electrode (Sentek, UK) with LiCl in ethanol filling solution. These were calibrated using aqueous pH buffers (CRMs produced in-house, CMI) versus their respective internal RE (Ag/AgCl). The signal of the GE portion of the combination electrodes were isolated using a BNC to banana plug converter. Potential difference was measured using a Potentiostat Biologic SP200. These electrodes (Thermofisher & Sentek) were tested in the differential potentiometry configuration using the CMI aqueous pH buffers. No change in signal was recorded regardless of the buffer species. It was concluded these combination electrodes are not suitable for differential potentiometry measurement isolating for the signal of the GE components, and experiments were discontinued.

SCGEs were calibrated versus a RE (Elektrochemicke Detektory, Czech Republic) consisting of a Hg/Hg_2Cl_2 reference with saturated KCl (aq) filling solution in aqueous pH buffers (4.000, 7.000, and 9.180, ± 0.02, CMI CRMs). Water-solvent solutions were prepared gravimetrically (wt% basis) using methanol (≥ 99.9 % for HPLC, Sigma Aldrich). Data was acquired for 2 h at 10 s intervals. Solutions used in constructing the $pH_{abs}^{H_2O}$ ladder included: three aqueous pH buffers (4.000, 7.000, and 9.18), and 3 water-methanol (25%, 50%, and 75% methanol).

Data was analyzed by averaging a 30 min interval between 1800 s and 3600 s (averaging), or by extrapolating this data to time zero (extrapolated). $pH_{abs}^{H_2O}$ ladders were constructed using both data analysis methods, using aqueous pH buffers 4.000 and 7.000 as anchoring buffers.

DFM

Measurements were performed in a thermostated cell (25.0 °C), using two commercial GE (half-cells) (E11M001, Radiometer Medical Aps, Denmark). These were calibrated using aqueous pH buffers (4.005, 7.000, and 9.18 at 25 °C, ± 0.01, Hach Lange) versus a RE (Radiometer Analytical REF201 red rod reference electrode, Hach Lange) consisting of a Ag/AgCl reference with saturated KCl (aq) filling solution. Calibration was performed without thermostating, at room temperature (~ 22.5 °C). Potential difference was measured using a high impedance analyzer (IM6eX, Zahner-Elektrik GmbH & CoKG, Germany). Additionally, two Polylyte Plus (Hamilton) combination pH electrodes were tested in the differential potentiometry configuration using the aqueous pH buffers. No change in signal was recorded regardless of the buffer species. It was concluded these combination electrodes are not suitable for differential potentiometry measurement isolating for the signal of the GE components, and experiments were discontinued.

Data on the half-cell GEs was acquired for 1 h at 10 s intervals. Solutions used in constructing the $pH_{abs}^{H_2O}$ ladder included: three aqueous pH buffers (4.005, 7.000, and 9.18), three water-ethanol solutions (50%, 70%, and

100% ethanol), 23%: 76% water-acetonitrile, and 23%: 76 % water-methanol. Water-solvent solutions were prepared gravimetrically (wt% basis) using anhydrous ethanol (99.97% purity, VWR), acetonitrile (99% purity, VWR), and methanol (99.9% purity, Merck). The solvents were used as purchased without any further treatment.

Data was analyzed by averaging the final 30 min of data acquired (averaging), or by extrapolating this data to time zero (extrapolated). $pH_{abs}^{H_2O}$ ladders were constructed using both data analysis methods, using aqueous pH buffers 4.005 and 7.000 as anchoring buffers.

SCGE (Electrode A), which were calibrated against the RE in aqueous pH buffers, were fitted into loose fitting lids and placed into each measurement pot. Solutions used in constructing the $pH_{abs}^{H_2O}$ ladder included: two aqueous pH buffers (4.005, and 7.000), two water-ethanol (70%, and 100% ethanol), two water-acetonitrile (76% and 100% acetonitrile), and two water-methanol solutions (76%, and 100% methanol).

IPQ

Measures were performed in a thermostated cell (25.0 °C), using two commercial GE (half-cells) (Metrohm 6.0150.100). These were calibrated using aqueous pH buffers (4.006, 6.865, and 10.012 at 25 °C, ±0.02, Mettler InLab Solutions) versus a RE (Metrohm 6.0729.108) consisting of a Ag/AgCl reference with a saturated LiCl ethanol filling solution. Potential difference was measured using a Keithley 6514 electrometer. Data was acquired for 1 h at 5 s intervals. Solutions used in constructing the $pH_{abs}^{H_2O}$ ladder included: three aqueous pH buffers (4.006, 6.865, and 10.012), 5 water-ethanol solutions (10%, 30%, 50%, 70%, and 100% ethanol), and two water-acetonitrile solutions (17% and 44% acetonitrile). Water-ethanol solutions were prepared gravimetrically (wt% basis) using anhydrous ethanol (~ 100 %, Carlo Erba) and UPW.

Data was analyzed by averaging the final 30 min of data acquired (averaging), or by extrapolating this data to time zero (extrapolated). $pH_{abs}^{H_2O}$ ladders were constructed using both data analysis methods, using aqueous pH buffers 4.006 and 6.865 as anchoring buffers.

SCGE, which were calibrated against the RE (Metrohm 6.0733.100) consisting of a Ag/AgCl reference with 3 M KCl (aq) filling solutions, in aqueous pH buffers (4.00, 7.00, and 9.00, Metrohm), were fitted into loose fitting lids and placed into each measurement pot. Solutions used in constructing the $pH_{abs}^{H_2O}$ ladder included: four aqueous pH buffers (4.00, 4.01, 6.87, and 7.000), five water-ethanol (10%, 20%, 50%, 70%, and 100% ethanol), and three water-acetonitrile (17%, 44% and 100% acetonitrile), Water-organic solutions were prepared gravimetrically (wt% basis) using anhydrous ethanol, acetonitrile (≥ 99.5%, AnalaR NORMAPUR) and UPW.

PTB

Measures were performed in a thermostated cell (25.0 °C), using two commercial GE (combination electrodes) (Metrohm 6.0269.100) with 3 M KCl (aq) filling solution. These were calibrated using aqueous pH buffers (4.01, 7.00, and 9.00 at 25 °C, ± 0.02, Certipur) versus a RE (Metrohm 6.075.100) with 3 M KCl (aq) filling solution. Potential difference was measured using a Keithley B2987A Electrometer/High Resistance Meter with Quick IV Measurement Software. Data was acquired for 1 h at 10 s intervals. Solutions used in constructing the $pH_{abs}^{H_2O}$ ladder included: two aqueous pH buffers (4.01, and 7.00), 7 water-ethanol solutions (10%, 30%, 50%, 70%, 80%, 90%, and 100% ethanol), 4 water-acetonitrile solutions (17%, 44%, 76% and 100% acetonitrile), and 4 water-methanol (17%, 44%, 76%, and 100% methanol). Water-solvents solutions were prepared gravimetrically (wt% basis) using anhydrous ethanol (≥ 99.9%, EMSURE, and ≥ 99.5%, EMPARTA, Merck), acetonitrile (99.95%, Th. Geyer), and methanol (99.9%, Th. Geyer), and de-ionized water (DI).

Data was analyzed by averaging for the data acquired between 1800 s and 3600 s of each measurement (averaging), or by extrapolating this data to time zero (extrapolated). $pH_{abs}^{H_2O}$ ladders were constructed using both data analysis methods, using aqueous pH buffers 4.01 and 7.00 as anchoring buffers. The averaging method, and associated data has previously been presented in [16] (combined electrode, Figure 3 there within).

SCGE, which were calibrated against the RE in aqueous pH buffers, were fitted into screw caps with centric bore and placed into each measurement pot. Data was acquired for 1 h at 10 s intervals. The final 30 min of data were averaged to provide the measurement potential differences.

UT

Measures were performed in a thermostated cell (25.0 °C), using two SCGE, which were calibrated using aqueous pH buffers (4.00, 7.00, and 10.00 at 25 °C, ±0.02, Honeywell/Fluka) versus a RE (Radiometer K401) with a saturated calomel electrode reference. Potential difference was measured using a Gamry Reference 3000 potentiostat and Metrohm 713 pH meter. Data was acquired for 1 h at 5 s intervals. The final 30 min of data were averaged to provide the measurement potential differences. Solutions used in constructing the $pH_{abs}^{H_2O}$ ladder included: aqueous pH buffers (4.01, 6.87, 7.00, and 10.01), 50%: 50% water-ethanol, 2 water-acetonitrile solutions (76% and 100% acetonitrile), and 3 water-methanol (44%, 76% and 100% methanol). Water-solvents solutions were prepared gravimetrically (wt% basis) using anhydrous ethanol (absolute, Honeywell Riedel-de-Haën), acetonitrile (CHROMASOLV, ≥ 99.9%, for LC-MS Honeywell Riedel-de-Haën), and methanol (CHROMASOLV, for HPLC, ≥ 99.9%, Honeywell Riedel-de-Haën), and UPW.

Commercial electrodes

Table S1. BFKH (Electrode B) calculated $pH_{abs}^{H_2O}$ values using two data extraction methods: averaging of over several hours of data, and extrapolation to time zero. Aqueous pH buffers (6.865 and 4.008) used as anchoring buffers. Data presented for aqueous buffers and water-ethanol solutions.

**Solution**	**Calculated** $pH_{abs}^{H_2O}$	
	**Averaging**	**Extrapolated**
80% MeOH	9.135	9.169
pH 9.18	9.008	8.982
50% MeOH	7.870	7.889
20% MeOH	6.790	6.819
pH 6.865		
pH 4.008		

Table S2. DFM (Electrode G) calculated $pH_{abs}^{H_2O}$ values using two data extraction methods: averaging of final 30 min of data, and extrapolation to time zero. Aqueous pH buffers (7.00 and 4.005) used as anchoring buffers. Data presented for aqueous buffers and water-ethanol solutions.

**Solution**	**Calculated** $pH_{abs}^{H_2O}$	
	**Averaging**	**Extrapolated**
pH 9.18	9.270	9.250
100% EtOH	8.380	8.380
70% EtOH	7.895	7.895
50% EtOH	7.125	7.049
pH 7.00		
pH 4.005		

Table S3. DFM (Electrode G) calculated $pH_{abs}^{H_2O}$ values using two data extraction methods: averaging of final 30 min of data, and extrapolation to time zero. Aqueous pH buffers (7.00 and 4.005) used as anchoring buffers. Data presented for aqueous buffers, water-(ethanol, acetonitrile, methanol) solutions.

Solution	Calculated $pH_{abs}^{H_2O}$	
	Averaging	Extrapolated
pH 9.18	9.201	9.187
76% MeCN	9.390	9.366
70% EtOH	8.130	8.100
50% EtOH	7.262	7.201
pH 7.00		
76% MeOH	6.354	6.316
pH 4.005		

Table S4. IPQ (Electrode H) calculated $pH_{abs}^{H_2O}$ values using two data extraction methods: averaging of final 30 min of data, and extrapolation to time zero. Aqueous pH buffers (6.865 and 4.006) used as anchoring buffers.

Solution	Calculated $pH_{abs}^{H_2O}$	
	**Averaging**	**Extrapolated**
pH 10.012	9.952	9.969
100% EtOH	7.912	7.948
pH 6.865		
70% EtOH	6.758	6.745
17% MeCN	6.212	6.172
30% EtOH	6.205	6.179
44% MeCN	6.202	6.131
50% EtOH	5.848	5.855
10% EtOH	5.084	5.079
pH 4.005		

Table S5. PTB (Electrode F) calculated $pH_{abs}^{H_2O}$ values using two data extraction methods: averaging 30 min of data, and extrapolation to time zero. Aqueous pH buffers (7.00 and 4.01) used as anchoring buffers.

Solution	Calculated $pH_{abs}^{H_2O}$	
	**Averaging**	**Extrapolated**
100% MeOH	8.46	8.75
76% MeOH	7.83	7.86
76% MeCN	7.46	7.57
90% EtOH	7.17	7.19
80% EtOH	7.16	7.22
70% EtOH	7.02	6.99
pH 7.00		
100% MeCN	6.93	6.98
100% EtOH	6.81	6.62
44% MeOH	6.78	6.79
50% EtOH	6.65	6.65
44% MeCN	6.43	6.51
30% EtOH	6.18	6.17
17% MeCN	5.90	5.93
17% MeOH	5.83	5.85
10% EtOH	5.53	5.55
pH 4.01		

Additional information acquired at PTB include published [16] results on 50 wt% and 80 wt% ethanol-water solutions. Using the averaging data analysis method, for 50 wt% ethanol, $pH_{abs}^{H_2O}$: 6.91, 7.80, and 7.38 for electrodes H, I, and J. For 80 wt% ethanol, $pH_{abs}^{H_2O}$: 8.91, 8.06, and 9.01 for electrodes H, I, and J.

SCGE (Electrode A)

Table S6. CMI (Electrode A) calculated $pH_{abs}^{H_2O}$ values using two data extraction methods: averaging of over 30 min of data, and extrapolation to time zero. Aqueous pH buffers (7.00 and 4.000) used as anchoring buffers. Data presented for aqueous buffers and water-methanol solutions.

Solution	Calculated $pH_{abs}^{H_2O}$	
	**Averaging**	**Extrapolated**

pH 9.18	9.178	9.150
pH 7.000		
50% MeOH	6.907	6.876
75% MeOH	6.489	6.464
25% MeOH	6.228	6.182
pH 4.000		

Table S7. DFM (Electrode A) calculated $pH_{abs}^{H_2O}$ values using two data extraction methods: averaging of final 30 min of data, and extrapolation to time zero. Aqueous pH buffers (7.00 and 4.005) used as anchoring buffers. Data presented for aqueous buffers, water-(ethanol, acetonitrile, methanol) solutions.

**Solution**	**Calculated** $pH_{abs}^{H_2O}$	
	**Averaging**	**Extrapolated**
76% MeCN	9.282	9.390
100% EtOH	7.830	7.422
70% EtOH	7.827	7.819
pH 7.000		
100% MeOH	6.713	6.562
100% MeCN	6.407	5.772
76% MeOH	6.373	6.375
pH 4.005		

Table S8. IPQ (Electrode A) calculated $pH_{abs}^{H_2O}$ values using two data extraction methods: averaging of final 30 min of data, and extrapolation to time zero. Aqueous pH buffers (6.865 and 4.006) used as anchoring buffers.

Solution	Calculated $pH_{abs}^{H_2O}$	
	Averaging	Extrapolated
100% EtOH	7.99	7.97
70% EtOH	7.81	7.75
50% EtOH	7.53	7.53
44% MeCN	7.24	7.22
pH 6.87		
20% EtOH	6.43	6.45
17% MeCN	6.33	6.39
10% EtOH	6.03	6.06
pH 4.01	3.86	3.85
pH 4.006		

Table S9. PTB (Electrode A) calculated $pH_{abs}^{H_2O}$ values using data extracted by averaging 30 min of acquired data. Values are given for two different purities of ethanol employed in solution preparation: 99.9% (left column) and 99.5% (right column). Aqueous pH buffers (10.01, 9.18 and 6.87) used as anchoring buffers.

**Solution**	**Calculated** $pH_{abs}^{H_2O}$			
	**Averaging**		**Extrapolated**	
	**99.9 % EtOH**	**99.5 % EtOH**	**99.9 % EtOH**	**99.5 % EtOH**
pH 10.01				
pH 9.18				
90% EtOH	9.57		9.50	
100% EtOH	9.41		9.26	
80% EtOH	9.12	7.84	9.09	7.80
100% MeOH	8.65		8.58	
70% EtOH	8.48		8.46	
100% MeCN	8.21		7.65	
76% MeCN	8.20		8.24	
76% MeOH	8.13		8.11	
50% EtOH	7.44	6.82	7.47	6.85
pH 7.00	7.00		7.00	
pH 6.87				
44% MeOH	6.99		6.95	
44% MeCN	6.78		6.73	
40% EtOH	6.74		6.72	
30% EtOH	6.57		6.59	
25% MeOH	6.43		6.35	
25% MeCN	6.30		6.32	
20% EtOH	6.29		6.30	
17% MeCN	6.23		6.23	
17% MeOH	6.20		6.21	
10% EtOH	6.00		5.99	
pH 4.01	4.01		4.01	

Table S10. UT (Electrode A) calculated $pH_{abs}^{H_2O}$ values from averaging 30 min of data for the potential differences measurements. Aqueous pH buffers (10.01, 7.00, and 4.01) were used as anchoring buffers.

**Solution**	**Calculated** $pH_{abs}^{H_2O}$	
	**Averaging**	**Extrapolated**
pH 10.01		
100% MeCN	9.78	8.78
100% MeOH*	8.96	8.94
100% MeOH*	6.70	6.62
76% MeOH	8.71	8.67
76% MeCN	8.69	8.75
50% EtOH	8.12	8.08
44% MeOH	7.94	7.88
pH 7.00		
pH 4.01		

* Different bottles
